# Establishing a simple perfusion cell culture system for light-activated liposomes

**DOI:** 10.1038/s41598-023-29215-6

**Published:** 2023-02-04

**Authors:** Eija Ilvesroiha, Patrick Lauren, Natsumi Uema, Kanako Kikuchi, Yuuki Takashima, Timo Laaksonen, Tatu Lajunen

**Affiliations:** 1 Division of Pharmaceutical Biosciences, Faculty of Pharmacy, University of Helsinki, 00790 Helsinki, Finland; 2 Department of Formulation Sciences and Technology, Tokyo University of Pharmacy and Life Sciences, Tokyo, 192-0392 Japan; 3 Faculty of Engineering and Natural Sciences, Tampere University, 33720 Tampere, Finland; 4 Faculty of Health Sciences, University of Eastern Finland, 70600 Kuopio, Finland

**Keywords:** Drug delivery, Nanomedicine, Biological models

## Abstract

The off-target effects of light-activated or targeted liposomes are difficult to distinguish in traditional well plate experiments. Additionally, the absence of fluid flow in traditional cell models can lead to overestimation of nanoparticle uptake. In this paper, we established a perfusion cell culture platform to study light-activated liposomes and determined the effect of flow on the liposomal cell uptake. The optimal cell culturing parameters for the A549 cells under flow conditions were determined by monitoring cell viability. To determine optimal liposome treatment times, particle uptake was measured with flow cytometry. The suitability of commercial QuasiVivo flow-chambers for near-infrared light activation was assessed with a calcein release study. The chamber material did not hinder the light activation and subsequent calcein release from the liposomes. Furthermore, our results show that the standard cell culturing techniques are not directly translatable to flow cultures. For non-coated liposomes, the uptake was hindered by flow. Interestingly, hyaluronic acid coating diminished the uptake differences between the flow and static conditions. The study demonstrates that flow affects the liposomal uptake by lung cancer cell line A549. The flow also complicates the cell attachment of A549 cells. Moreover, we show that the QuasiVivo platform is suitable for light-activation studies.

## Introduction

Dynamic cell culturing platforms that combine fluid flow and the possibility to cultivate several distinct cell lines have been under extensive investigation during the last two decades due to their potency in replicating the complex human body phenomena. Especially after the role of cancer microenvironment to cancer cell behavior was understood^1^, the interest in creating biomimicking cancer cell cultures that consider microenvironmental cues has increased. These biomimicking cell culture platforms aim to provide more in vivo-like environment compared to traditional in vitro methods. Furthermore, the anticancer drug resistance increases in “organ-on-a-chip” cell culture models when compared to traditional well plate assays^2^. This replicates the clinical findings of anticancer treatments more appropriately than what we can see on the normal well plate assays. Utilizing these models should increase the in vitro–in vivo translatability, and thus, lead to better research outcomes.

Nanoparticle research has also faced similar translatability problems. Although multiple liposomal and other nanoparticle drug carriers have shown great efficiency in preclinical studies, they fail to replicate the same effect in clinical studies^3^. Various reasons for the poor translation have been proposed^4,5^. Within in vitro studies, e.g., the lack of immune system^6^ and the sedimentation of nanoparticles^7^ have been considered as possible factors. Sedimentation causes particles to lay on top of the cells, increasing the likelihood of interaction between the nanoparticles and cells. The long contact time causes bias both in toxicity and efficiency studies, as normally the nanoparticles would be moving along with the interstitial fluid flow, which challenges them from reaching their target sites.

Therefore, dynamic cell culture systems will be relevant for studying nanoparticles in the future. The fluid flow and shear stress generated by those systems have shown to affect nanoparticle uptake^8–12^, indicating that the flow is indeed an important factor affecting the use of nanocarriers (e.g. liposomes and micelles) in drug delivery. Potentially, the capability of targeted nanoparticles could be better established within a dynamic cell culture system, where flow challenges the nanoparticles to find their target region. As a particular targeting ligand, in the current study we are interested in hyaluronic acid (HA, also referred to as hyaluronan), which can be conjugated onto liposome surface and utilized as a targeting moiety directed towards cancers^13^. HA binds to cell surface receptor CD44, which is overexpressed in several invasive tumors and can lead to enhanced nanoparticle uptake^14–16^.

Interstitial fluid flow is known to differ between healthy and neoplastic tissues^17^. It is responsible for nutrient and metabolite transportation in the cellular level. In addition to its important role in maintaining homeostasis, its movement creates shear stress on the cell surface. The shear stress is known to affect tumor proliferation and apoptosis^18^, epithelial–mesenchymal transition^19^, behavior of cancer stem cells^20^, and metastasis ^21^. Thus, this mechanistical force is an essential factor in the functioning of healthy and neoplastic cells. Such flow and its effects on nanoparticle uptake is excluded from traditional static cell cultures studies but can be studied with dynamic models.

Liposomes are nanoparticles mainly consisting of phospholipids. The phospholipid layer encloses an aqueous phase, creating lipophilic and lipophobic environments. Light-sensitive liposomes can be externally triggered with a laser. They contain a sensitizer molecule, i.e. a dye, which disrupts the normal liposomal biphasic structure after irradiation at a specific wavelength, leading to drug release^22^. As the target location of the laser beam can be predetermined, e.g., towards a tumor, the release is time and place specific. However, even drug delivery systems like these cannot perfectly regulate the drug release as liposomes do not completely prevent the drug action on healthy tissues. Due to the phagocytic behavior of the immune system and nonspecific liposomal uptake, healthy tissues are also exposed to the drug and side effects.

The traditional well plate experiments do not offer a representational setting to study the off-target effects of light-activated liposomes. Determining these effects requires a system, where the effects of light activation on on-target and off-target tissues can be studied within the same system but analyzed separately. For example, the on and off-target tissues can be cultivated within separate chambers, which are connected by perfusing media. This allows the liposomes and target cells to be irradiated in one chamber but not in the other. The perfusing media transports the non-activated liposomes and part of the released drug also to off-target cells, letting us to examine the consequences of light activation on off-target cells. Although determining the off-target effects of light-activated liposomes is not the focus of the present study, one should keep in mind that the resulting system can be examined for these experiments. We will focus on the off-target aspect in our future studies.

In this study, we characterized and optimized a light-activation add-on functionality to a commercial dynamic cell culture system, QuasiVivo^®^, to study light-activated liposomes. QuasiVivo^®^ is connected to a peristaltic pump, which creates a flow that resembles the natural interstitial flow at the cell surfaces. The ensuing shear stress in turn affects the cell behavior, as it has been seen in other QuasiVivo^®^ studies^23–26^. Multiple chambers can be connected within one and the same system, creating the possibility to include several cell lines—cultured in separate areas—within the same system. The aim of the study was to optimize QuasiVivo as a novel cell culture tool for studying light-activated liposomes. In our study, the suitability of QuasiVivo chamber material for light activation studies was determined with calcein release assay, the cell viability under multiple flow rates were assessed with AlamarBlue and Live/Dead assays, and finally, the intensity of liposomal uptake with and without hyaluronic acid coating in the dynamic and static conditions were examined with flow cytometry.

## Results

### Light activation through QuasiVivo chambers

**Figure 1:**
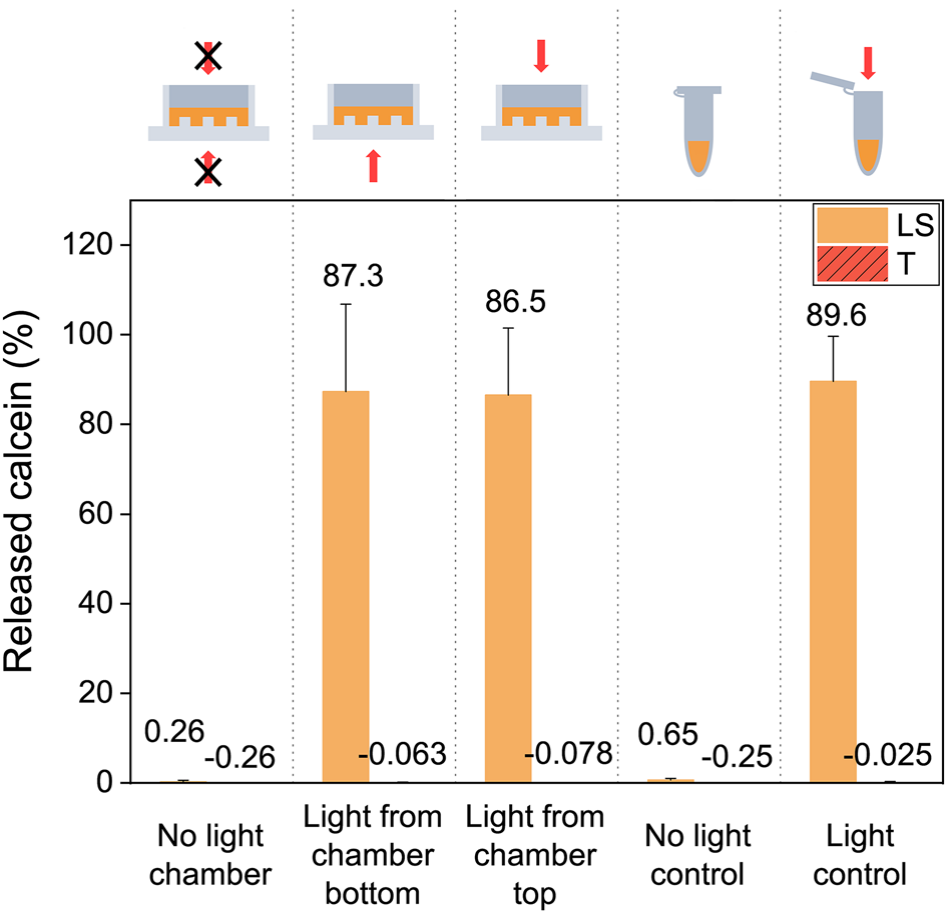
Calcein release from light-sensitive (LS) and traditional liposomes (T). For non-irradiated control samples, the calcein release was measured when the liposomal samples were stored within chambers (+ 37 °C) and in Eppendorf tubes (+ 4 °C). For the irradiated samples, the irradiation was performed from the bottom of the chamber (through the chamber material, + 37 °C), from the top of the chamber (without chamber lid on, + 37 °C), and the “light control” was irradiated within Eppendorf tubes, as previously described^27^. Mean ± s.d., n = 3.

To study external light-activation within QuasiVivo chambers, a calcein release study from liposomes was conducted (Fig. 1). Calcein release after irradiation showed to be similar when the light-activated liposomes were irradiated with (87.3 ± 19.5%) and without (86.5 ± 14.9%) the chamber material in between the laser and the liposomes (Fig. 1). In contrast, the calcein leakage from the light-activated liposomes was minimal without irradiation (0.3 ± 0.3% and 0.7 ± 0.4%, for liposomes activated within chambers and Eppendorf control tubes, respectively). The passive leakage from traditional liposomes was unaffected by irradiation in any setting.

### The dynamic cell culture of A549 lung cancer cells

**Figure 2:**
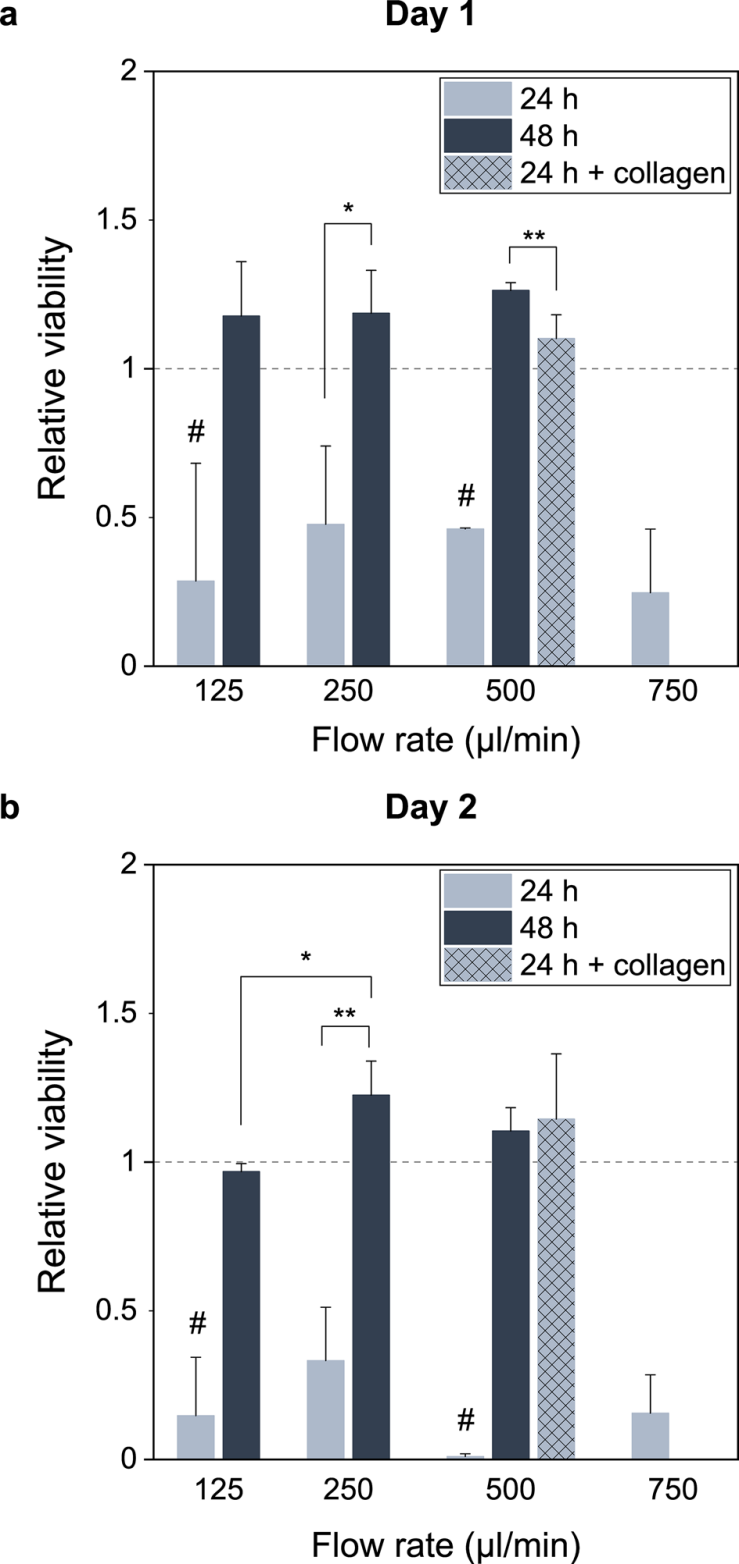
The relative viability of A549 cells cultured under flow, normalized to statically grown cells (controls of each experiment). The cells were let to attach for 24 or 48 h onto a non-coated coverslip or a collagen-coated coverslip. The graphs present the cell viabilities assessed with AlamarBlue assay after 1 (**a**) and 2 (**b**) days of culture inside the QuasiVivo system (mean ± s.d., each n includes the mean viability of two chambers from the same system). ^#^n = 2; *p < 0.05; **p < 0.01.

**Figure 3:**
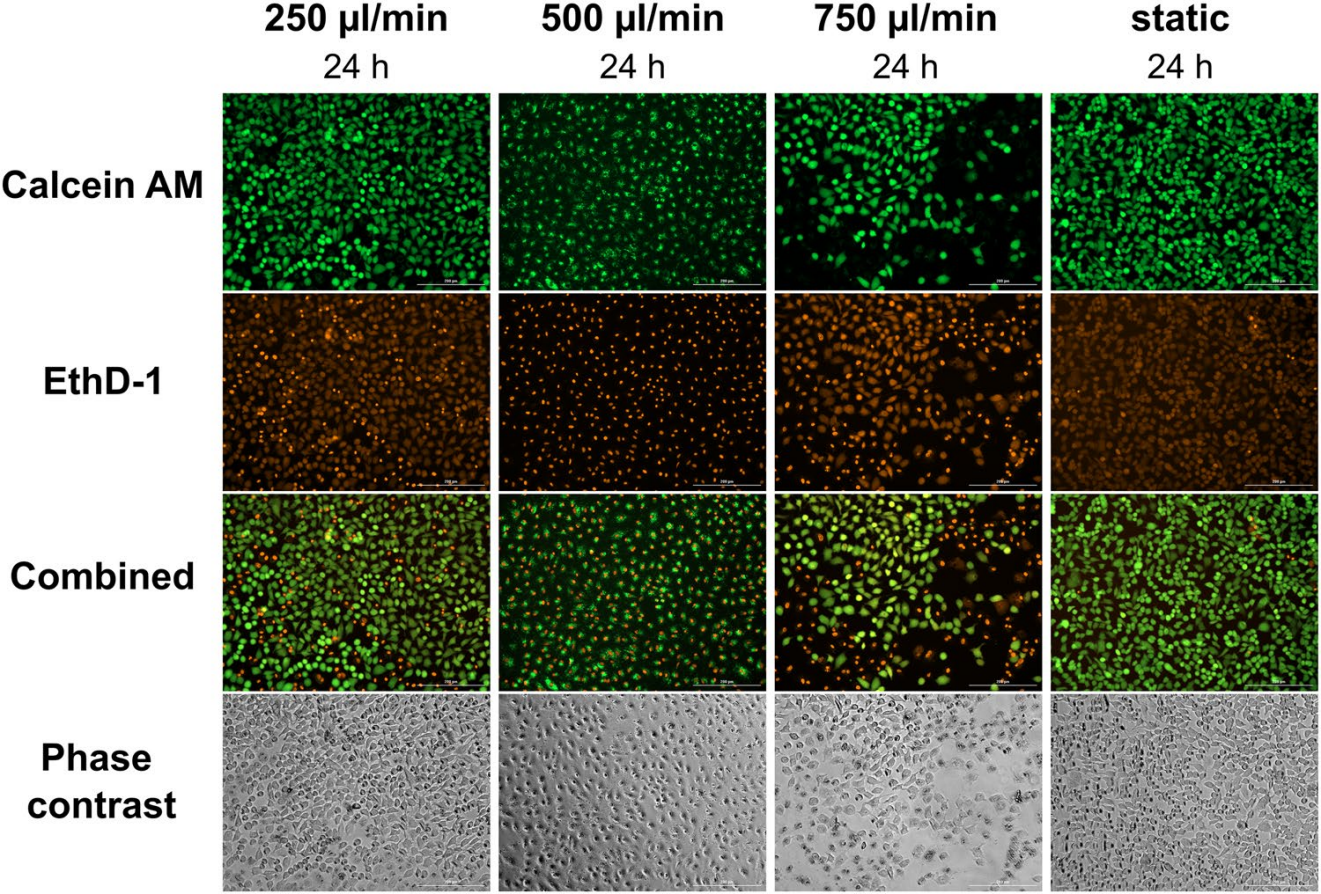
The viability of A549 cells under 250, 500, and 750 µl/ml flow, when the cells adhered on glass coverslips for 24 h. The cells were stained with Live/Dead viability/cytotoxicity assay. Calcein AM (green) shows alive cells and EthD-1 (ethidium homodimer-1) (orange) shows dead cells. With 500 µl/min flow rate, most of the cells seemed desiccated, which could explain the illogical decrease in viability after 2 days compared to 250 and 750 µl/min flow rates (X10, scale bar shows 200 µm).

Cell viability was examined after cultivation under different flow rates to optimize the culturing conditions for A549 cells (Fig. 2). The flow rates were selected to be close to 500 µl/min (125–750 µl/min), as the microenvironment generated by 500 µl/min (shear stress 1.01 × 10^–5^ Pa and flow speed 1.43 µm/s, calculated with the mathematical formula presented by Mazzei et al.^28^) resembles the in vivo relevant cell microenvironment^29^. For the different flow rates, the shear stress and flow speed values are 3.35 × 10^–6^ Pa and 0.455 µm/s (for the flow rate of 125 µl/min), 5.6 × 10^–6^ Pa and 0.78 µm/s (for 250 µl/min), and 14.6 × 10^–6^ Pa and 2.08 µm/s (for 750 µl/min). However, the observed viability values were lower under these conditions (Figs. 2a, 3). Increasing the flow rate to 750 µl/min decreased the viability even more when compared to other flow rates. By visual observation it was possible to see that the coverslips contained cell-free areas. This led to a conclusion that the cells might not properly adhere onto the coverslips and were carried off by the flow. Thus, the attachment time was increased from 24 to 48 h.

**Figure 4:**
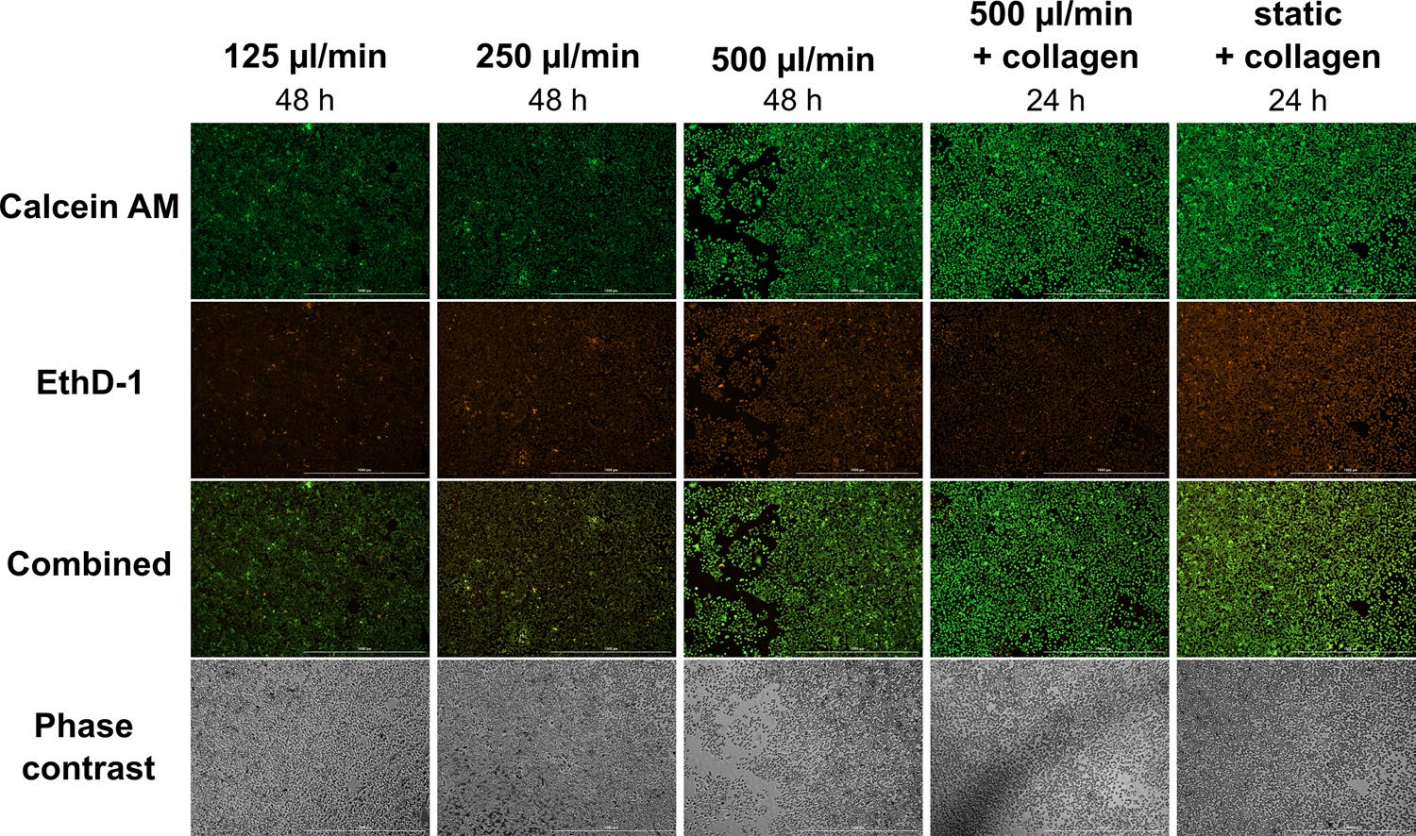
The viability of A549 cells under 125, 250, and 500 µl/min, when the cells were let to attach either onto glass coverslip for 48 h or onto collagen coated coverslip for 24 h. The cells were stained with Live/Dead viability/cytotoxicity assay. Calcein AM (green) shows alive cells and EthD-1 (ethidium homodimer-1) (orange) shows dead cells (X4, scale bar shows 1000 µm).

Increasing the attachment time from 24 to 48 h markedly increased the cell viability (Figs. 2, 3, 4). For the flow rate of 250 µl/min, the relative viability increased from 0.33 ± 0.18 to 1.22 ± 0.11 (mean ± s.d., p = 0.002). Also, the number of cells stained as “dead” in Live/Dead analysis decreased (Figs. 3, 4). Thus, the longer attachment time seemed to resolve the viability problem. The cell viability was similar for all the flow rates on day 1 (Fig. 2a). On day 2, a statistically significant difference in cell viability was recorded between cells grown under 125 µl/min and 250 µl/min (p = 0.02). Moreover, the cells cultured under 250 µl/min and 500 µl/min flow rate yielded slightly higher viabilities compared to statically cultured cells on day 2 (1.22 ± 0.11 and 1.10 ± 0.08 vs. 1.00, respectively).

Finally, a collagen coating was added into the 24-h attachment time protocol to examine if a biomimicking surface would benefit the cell viability (Fig. 2). On day 2, the cell viability of samples that were cultured on collagen for 24-h were comparable to samples that were cultured for 48-h without collagen (p = 0.767). Overall, the cell viability was slightly higher when the cells were let to attach either onto collagen for 24 h or onto normal glass coverslips for 48 h before the cultivation in QuasiVivo.

### Liposomal uptake by A549 cells under dynamic fluid flow

**Figure 5:**
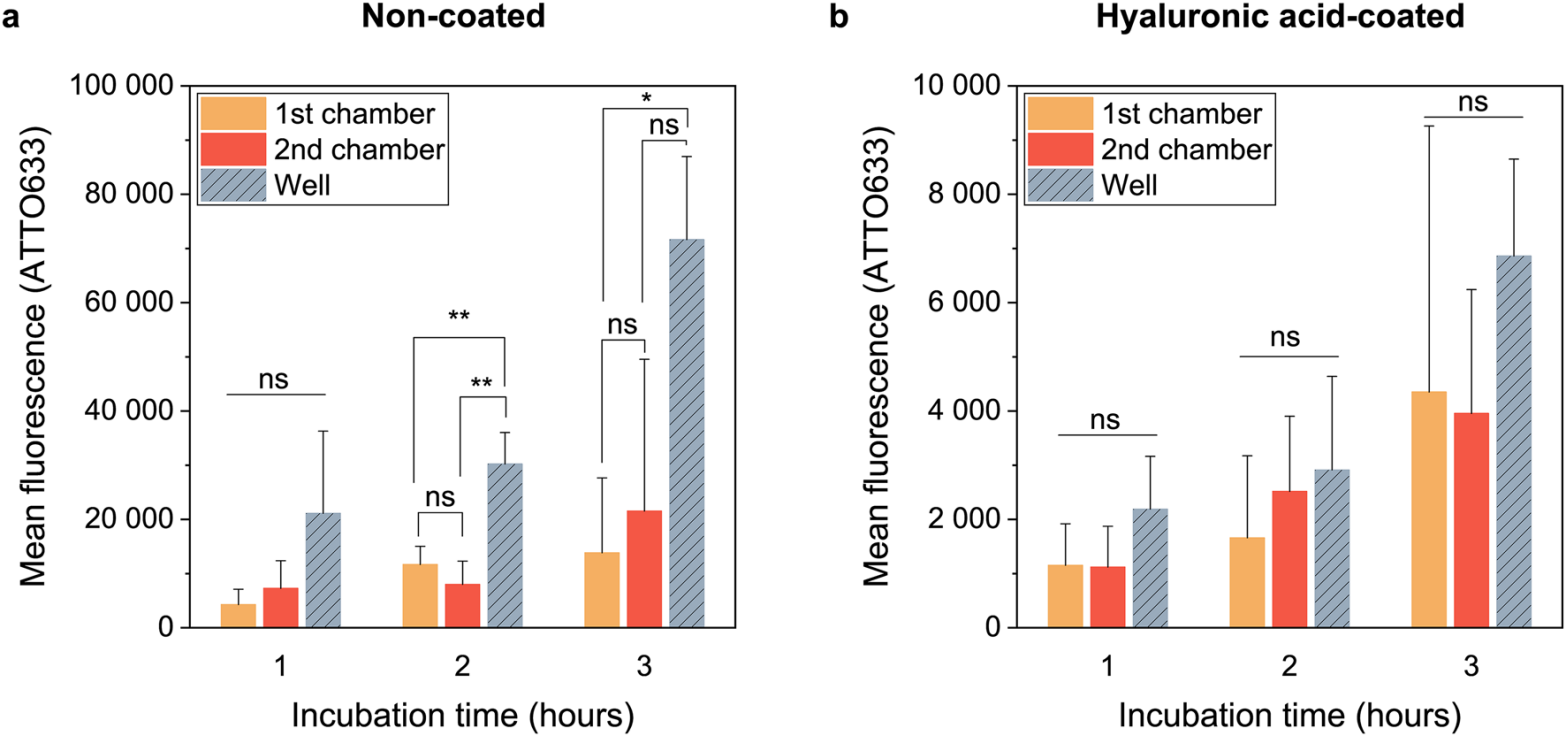
Liposomal uptake by A549 cells. Cells were incubated with ATTO633-labelled liposomes under static or dynamic conditions for 1, 2, or 3 h. The fluorescence intensity was measured with flow cytometry. (**a**) The mean fluorescence of cells incubated with non-coated liposomes. The cells were cultured either in QuasiVivo chambers or 24-well plates (mean ± s.d., n = 3). (**b**) The mean fluorescence of cells incubated with hyaluronic acid-coated liposomes. The cells were cultured either on QuasiVivo chambers or 24-well plates (mean ± s.d., n = 3). See Supplementary Fig. S3 for the fluorescence intensity histograms of each experiment. *p < 0.05, **p < 0.01, *ns* not significant.

To investigate the intensity of liposomal uptake under dynamic flow conditions, A549 cells were incubated with uncoated or HA-coated fluorescently stained liposomes within QuasiVivo. These were compared to static cultures on well plates (Fig. 5). For future studies with light-activated liposomes and their off-target effects, we were interested in examining whether the liposomal uptake between the first and second chamber would differentiate. Thus, the uptake within each chamber was studied individually.

For non-coated liposomes, the uptake between first and second chamber was similar at 1-, 2-, and 3-h time points (p = 0.919, p = 0.615, p = 0.888, respectively) (Fig. 5a). Likewise, the liposomal uptake was comparable between each QuasiVivo chamber and well plate after 1-h incubation (F_2,6_ = 2.756, p = 0.142). However, after the liposomes had been incubated with cells for 2 h, the uptake was significantly lower in QuasiVivo than in the well plate experiments (p = 0.006 for the first chamber, p = 0.003 for the second chamber). After 3-h incubation, the uptake was significantly lower (p = 0.029) in the first chamber when compared to the well plate, but the difference was not statistically significant (p = 0.051) between the second chamber and the well plates. According to these results, the liposomal uptake tends to be higher in traditional cell culture systems than in the QuasiVivo system.

For hyaluronic acid-coated liposomes, the uptake was similar between each chamber and the well at all the incubation time points (F_2,6_ = 1.578, p = 0.281 at 1 h; F_2,6_ = 0.513, p = 0.623 at 2 h; F_2,6_ = 0.686, p = 0.539 at 3 h) (Fig. 5b). Thus, the hyaluronic acid coating diminished the effect of flow on the liposomal cell uptake.

### The effect of HA-CD44 binding on the HA-liposome cell uptake

Additional experiment was conducted to determine the effect of CD44-binding on the cell uptake under static conditions. After 1-h incubation of HA-coated liposomes and A549 cells, which were pre-treated with free-HA, the liposomal uptake was about half compared to a control without free hyaluronic acid (7825 ± 774 vs. 14,322 ± 245, for free HA pre-treatment and control, respectively). However, the difference was diminished at 3-h incubation (25,982 ± 4500 vs. 32,505 ± 619, for free HA and control, respectively). In a separate experiment, PEG coating was shown to reduce the cellular uptake considerably showcasing the effect of steric stabilization of liposomes (Supplementary Fig. S4).

## Discussion

The poor in vitro–in vivo correlation in nanoparticle drug research is a current problem and needs to be solved to improve the predictability of nanoparticle behavior in vivo. Within this study, we describe the light activation add-on function development and optimization process of a commercial perfusion cell culture system, QuasiVivo^®^, for the purpose of studying the on- and off-target effects caused by light-activated liposomes. The topic featuring light-activation of liposomes within a dynamic cell culture system has not been studied before, therefore, we believe these results are valuable for other researchers as well.

First, the applicability of the system for light-activation was studied. QuasiVivo was optimized to be used for our light-activated liposome studies, therefore, its feasibility with the laser was assessed. The light that was transmitted through chambers was able to release calcein from the light-activated liposomes, and we conclude that it is possible to use QuasiVivo system for light activation studies. In further tests, we have also seen that this can be done also when cells are cultured on the chambers under flow. This, however, remains a subject to further in-depth studies in the future. Here we wanted to test the feasibility of the system for liposomes in general and the effect of flow for the particle uptake and cell viability.

The laser beam does not comprehensively cover the QuasiVivo chamber area meaning that part of the liposome sample is not triggered. This explains why the calcein release did not reach 100%. From a translational perspective, this partial release is actually more in vivo-relevant as one-time irradiation does not cover all the tissue, and the target area has to be irradiated multiple times from different positions to cover the whole target area.

As we assumed that the flow might challenge the uptake of liposomes, the incubation time for the liposomes had to be assessed to determine the optimal timepoint for the light activation studies. In line with our hypothesis, this study demonstrates that dynamic cell culture conditions decrease the liposomal uptake by lung cancer cell line A549. Moreover, the hyaluronic acid-coating mitigated this effect, as the uptake of hyaluronic acid-coated liposomes was similar in static and dynamic conditions. Additionally, we found that the same conditions, meaning the attachment time and collagen coating, did not work similarly for the dynamic and static cell cultures.

The cell uptake results contrast with the finding by Chen et al.^30^, as they found the flow of 5 ml/min to not affect the cell uptake of liposomes, when compared to static conditions. However, in their studies, the flow rate was chosen to resemble vascular flow rate, not interstitial flow rate as in our study. Additionally, they used endothelial cells, which are likely to behave differently under flow than epithelial cells. The liposomes both in our studies and in studies by Chen et al. were coated with polyethylene glycol, which is known to hinder the cell uptake of liposomes^31,32^, while making them more stable. The increased stability can decrease sedimentation, leading to decreased cell uptake in static cell cultures, which mitigates the uptake differences between static and dynamic conditions.

Previous studies have also described the cell uptake of other nanoparticles under flow rates, which resemble vascular flow^9,10^. Kang et al.^9^ conducted a cell uptake study with polystyrene nanoparticles. Shear stress of 0.5 dynes/cm^2^ (0.05 Pa) increased the uptake of polystyrene nanoparticles for three different cell lines, compared to static conditions. Furthermore, Kang et al.^10^ also studied the uptake of gelatin-oleic nanoparticles under flow. Unlike in the polystyrene nanoparticle study, the uptake of gelatin-oleic nanoparticles was similar under 0.5 dynes/cm^2^ and static conditions. But increasing the shear stress to 5 (0.5 Pa) and 50 dynes/cm^2^ (5 Pa) increased the uptake. In conclusion, these studies support the findings that flow affects the nanoparticle cell uptake, although the direction of the change seems to differ.

Earlier findings have shown hyaluronan coating to increase the liposomal uptake by CD44-expressing cells under static conditions^13,33^. For this reason, we chose the lung carcinoma cell line A549 as our model cell line, as it overexpresses CD44-receptors^34,35^. Additionally, in our future studies, our aim is to study the effects of anticancer drug-loaded light-activated liposomes with the QuasiVivo system, thus, a cancerous cell line that responds to the chosen anticancer drug was an important feature for the utilized cell line. Due to the CD44-receptor overexpression of A549 cells, the increased uptake seen in this study might be a consequence of CD44 involvement. However, since the actual CD44 levels are unclear it is not possible to make stronger assumptions about the involvement of CD44. Nonetheless, these results were in line with previous studies regarding the uptake of hyaluronic acid-coated liposomes.

As the fluids of our body are in constant movement, the fluid flow challenges the uptake of liposomes and other nanoparticles, as it is shown to do in vitro. Based on our results, we can clearly see that flow decreases the liposomal cell uptake. These results and previous findings highlight the need for more predictable in vitro systems. Improving cell cultures with a flow feature brings the cell culture one step closer to the cell microenvironment, shortening the in vitro–in vivo translatability gap seen in nanoparticle research. Although the traditional well plate assays facilitate high-throughput analysis, a dynamic cell culture is likely to save time in the long run. Dynamic cell cultures are viable tools to gain more knowledge e.g., prior entering animal studies with nanoparticles. Animal studies are time-consuming, expensive, and have ethical issues. Thus, they should be kept as minimum as possible. The usage of dynamic cell culture results may lead to more in vitro–in vivo translatable results and gives good preliminary data that helps to plan animal studies properly. Therefore, a dynamic cell culture system functions as an intermediate step before the initial animal studies.

The cell uptake experiment described here has some limitations. Although the total fluorescence of each cell is measured, the cell uptake experiment fails to assess the exact number of liposomes within each cell. Additionally, the technique does not consider the liposomal cycle into and out of the cell, leaving out of count the liposomes that have already entered and exited the cell. On the other hand, the fluorescent labels may stick into the cells upon entering, which could show as overly positive fluorescence results.

In the cell uptake study, the fluorescence intensity levels differed by tenfold between the non-coated and HA-coated liposomes. Stealth properties of HA-coating are likely to cause the difference, but other factors may also play a role. One possible option is that the fluorescence intensity was originally dissimilar between the formulations, either due to the formulation itself or due to the usage of different fluorescent dye batches. Thus, direct assumptions about the role of hyaluronic acid in the liposomal uptake should not be made only by comparing the liposomal uptake results between the non-coated and HA-coated liposomes presented here. Further studies are needed to confirm the causes.

Compared to the traditional static cell cultures, cells required either longer attachment time or a coating, when they were cultured under dynamic conditions. It is not surprising that cells do not adhere well onto non-coated glass coverslips, as glass does not serve multiple attachment sites for cells. Yamamoto et al.^36^ studied the attachment properties of murine fibroblast cells on different surfaces and the cells had the lowest cell adhesive shear strength on glass. In contrast, the cells cultured on collagen had approximately 3 times higher adhesive shear strength. Thus, it is likely that the flow causes shear stress on cell surface, straining them, and the weakly adhered cells detach from the glass coverslips. This hypothesis is supported by the fact that collagen decreased the time requirement for attachment.

Interestingly, the AlamarBlue viability assay showed slightly higher viability for QuasiVivo-grown cells compared to static. As AlamarBlue measures mitochondrial metabolic activity, the viability change could be due to e.g. increased cell metabolism or higher proliferation rate under flow, which would be interesting to examine further.

The effects of these small changes in the cell culture parameters, such as changing the attachment time and the cell culture environment from static to dynamic, underlines how important it is to optimize each of these microenvironmental factors for the cell line. Without optimization one cannot be sure that the cells remain viable during experiments.

Although the flow rate was chosen to be close to interstitial fluid flow values that have been reported in the literature, the methods to accurately assess the actual interstitial fluid flow and pressure are missing. Therefore, the simple assumption that the flow rate used here can be translated into the flow rate of the human body is not possible. Hopefully, the methods to accurately assess the small-scale fluid flow rates will improve in the near future, as the knowledge of the human body parameters would improve the feasibility of highly biomimicking organ-on-a-chip platforms.

Altogether, the results shown here and reported by other researchers give insights for the reasons behind the poor in vitro–in vivo translatability seen in nanoparticle research. The differences seen in the liposomal cell uptake between static and dynamic cultures support the use of dynamic cell culture methods in addition to the traditional well plate assays. According to our results, QuasiVivo is a suitable platform to utilize in the liposome research and to study externally triggered liposomes.

## Methods

### Materials and reagents

1,2-Dipalmitoyl-*sn*-glycero-3-phosphocholine (DPPC), 1,2-distearoyl-*sn*-glycero-3-phosphocholine (DSPC), 1-stearoyl-2-hydroxy-*sn*-glycero-3-phosphocholine (Lyso PC 18), and 1,2-distearoyl-*sn*-glycero-3-phosphoethanolamine-*N*-[methoxy(polyethylene glycol)-2000] (ammonium salt) (DSPE-PEG2000, 880120P) were purchased from Avanti^®^ Polar Lipids, Inc., USA. 1,2-Dioleoyl-*sn*-glycero-3-phosphoethanolamine labelled with ATTO633 (ATTO633-DOPE; 90077, Lot# BCCF5108) and indocyanine green (ICG; 1340009) were purchased from Merck. Another vial of ATTO633-DOPE (AD 633-161; Lot# SA07X26), which was used only for HA-coated liposome uptake study, was purchased from ATTO-TEC GmbH. 1,2-distearoyl-*sn*-glycero-3-phosphoethanolamine-*N*-hyaluronic acid (DSPE-HA) was prepared according to reported method^37^, and the DSPE-HA conjugation was confirmed with Fourier-transform infrared spectroscopy (Supplementary Fig. S5).

Collagen from rat tail (Sigma-Aldrich, C7661-5MG, Lot# SLCB3933) was diluted into 0.25% acetic acid solution to obtain a 0.1% collagen stock solution. The stock solution was further diluted into a 0.01% working solution.

Dulbecco’s Modified Eagle’s Medium with high glucose (DMEM) (Sigma-Aldrich, D6429-500ML, Lot# RNBJ7900) supplemented with 10% fetal bovine serum (Gibco™, Thermo Fisher Scientific) and 1% penicillin–streptomycin (Gibco™, Thermo Fisher Scientific) was used in the cell experiments. LIVE/DEAD™ Viability/Cytotoxicity kit for mammalian cells (Invitrogen™, USA) and AlamarBlue Cell Viability Reagent (Invitrogen™, USA) were purchased from Thermo Fisher Scientific, Inc (USA).

### QuasiVivo system characteristics

**Figure 6:**
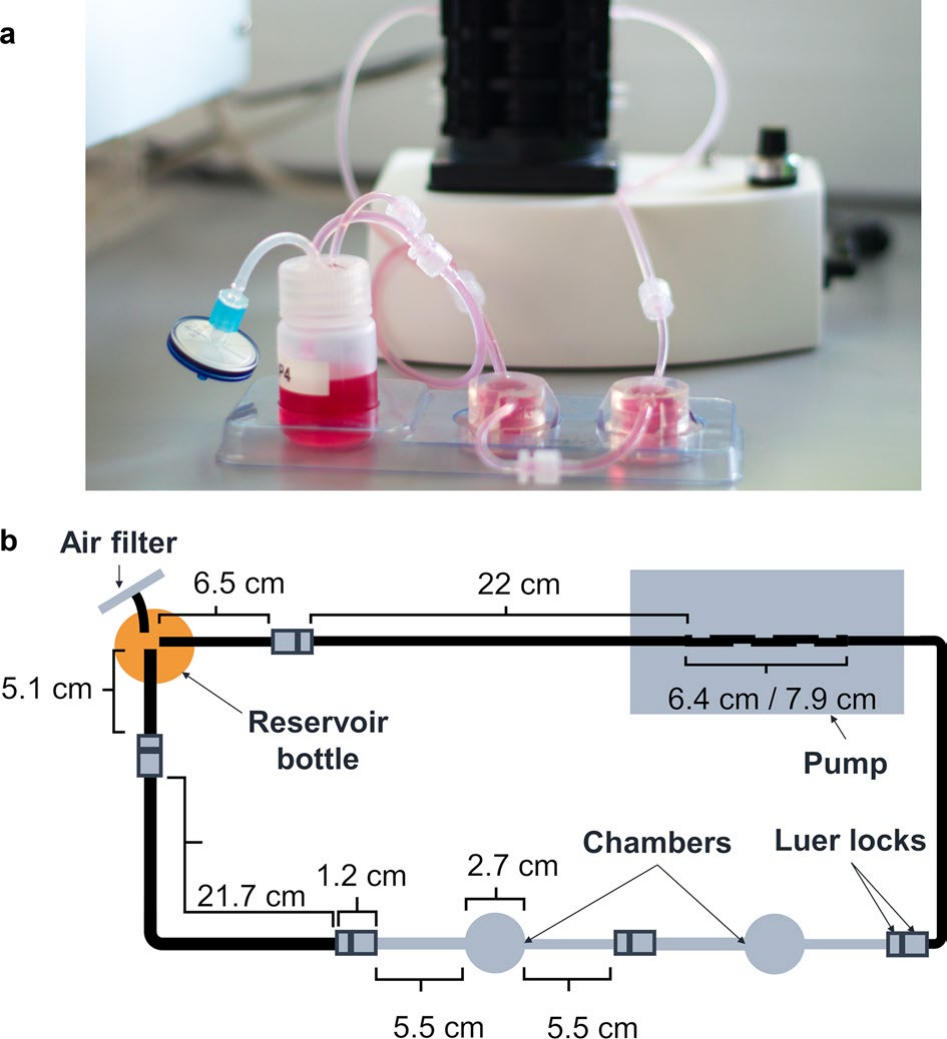
The QuasiVivo system. (**a**) The assembled QuasiVivo system. Medium moves from the reservoir bottle (on left) towards the pump, where the peristaltic movement is created. Then, it flows through the chambers, where cells are cultured, and returns to the reservoir bottle. The tubes are connected with luer locks. (**b**) Measures of QuasiVivo system. The pump tubing length depends on the used pump, PF22X0103 having longer tubes.

QuasiVivo system (Kirkstall Ltd, UK) contained a medium reservoir bottle (30 ml), air filter (0.2 µm PES; Corning 431229), two chambers (QV500), two inlet tubes (1/16 “), pump tubing, and one outlet tube (3/32″) (Fig. 6). Peristaltic flow was generated using Parker Polyflex 2- and 6-channel peristaltic pumps (PF22X0103 and PF-TP-601, Parker, USA). Cells were seeded on round glass coverslips (ø 12 mm, thickness 0.13–0.16 mm).

### Liposome preparation

Light-activated liposomes were prepared by a thin-film hydration method, followed by size-adjustment by extrusion, and purification by size-exclusion chromatography, similarly as described earlier^27^. Briefly, lipids (DPPC, DSPC, Lyso PC 18, DSPE-PEG2000) were dissolved in chloroform in molar ratios 75:15:10:4, respectively. Total lipid amount was 20.73 µmol. Chloroform was evaporated in a rotavapor, and lipids were rehydrated with HEPES buffer (0.5 ml; 20 mM HEPES, 140 mM NaCl, pH 7.4) that contained indocyanine green (ICG) (0.644 mg/ml) and/or calcein (60 mM, 280 mOsm, pH 7.4), depending on the liposome formulation. Indocyanine green is a light sensitizer molecule, which allows the cargo release within these light-activated liposomes. Lipid-buffer mixture was warmed in a water bath (63 °C) and mixed with a vortex mixer several times until the visible lipid layer had disappeared. To ensure the complete detachment of lipids, the sample was kept in the water bath for 20 min and mixed every five minutes. Afterwards, the formed polydisperse liposomes were extruded through a polycarbonate filter (0.1 µm pore size) 11 times with an extruder. Then, the sample was collected and immediately cooled down under running water. Lastly, the liposomes were purified with a column (1 cm × 20 cm) filled with Sephadex G50 (Cytiva, USA) and HEPES buffer. The final lipid concentration was 3.73 mM.

For flow cytometry experiments, fluorescently labelled liposomes without PEG-coating were fabricated with a similar protocol. The molar ratio for non-coated liposomes was 75:15:10:4:0.5 mol:mol (DPPC, DSPC, Lyso PC 18, DSPE, Atto633-DOPE, respectively) and the lipid film was hydrated with ICG-HEPES-buffer. The final lipid concentration was 3.75 mM. The liposomes were prepared and stored in dark.

The HA-coated liposomes were prepared with a modified protocol, due to the hydrophilicity of HA-conjugated DSPE. The molecular ratio of the lipids was 75:15:10:0.5:1 mol:mol (DPPC, DSPC, Lyso PC 18, Atto633-DOPE and DSPE-HA, respectively). A lipid thin-film was prepared of DPPC, DSPC, Lyso PC 18, and Atto633-DOPE. DSPE-HA was added at the hydration step; DSPE-HA was dissolved in HEPES buffer by warming and mixing the solution. To form a hydration buffer, the DSPE-HA solution was mixed with an ICG-HEPES mixture. The final lipid concentration was 3.65 mM. Methods of polyethylene glycol-coated liposomes uptake study under static conditions can be found in the Supplementary Information.

### Suitability of the QuasiVivo chambers for light activation studies

The suitability of the laser with QuasiVivo chambers was determined by assessing calcein release level from the liposomes. Calcein is a fluorescent, hydrophilic molecule, that self-quenches at high concentration levels. Thus, when calcein is not leaking from the liposome, the emission remains near zero^38,39^. The liposomes were irradiated (2000 mW, 808 nm, 3.2 W/cm^2^; ML6500, Modulight Oy, Finland) through the bottom of the chamber. Similarly, the laser was placed above the chamber and the liposomes were irradiated without the lid in between. As an additional control, the liposomes were irradiated inside ThermoMixer^®^ C (Eppendorf, Germany), where appropriate release is known to occur^25^. Passive release without light activation was also studied. The negative controls for both formulations were stored in the refrigerator.

The amount of released calcein was studied by assessing the fluorescence of calcein. To avoid self-quenching of calcein outside the liposomes, the samples were diluted in 1:12.5 ratio with HEPES-buffer. Each sample was measured with Varioskan LUX (493/518 nm, Thermo Fisher Scientific, USA). Afterwards, Triton™ X-100 (20 µl, 10%; MP Biomedicals, USA) was added to each sample, incubated for 2 min, and measured again. Triton™ X-100 disrupts the liposomal structure, releasing calcein from the inner liposomal phase. The relative calcein release was calculated with the following Eq. (1):

1 $$R=\frac{F-{F}_{0}}{{F}_{100}-{F}_{0}}\times 100\%,$$

where R is the relative calcein release, F is the initially measured fluorescence, F_0_ is fluorescence for the control sample, and F_100_ is the maximum fluorescence (the fluorescence measured after Triton™ X-100 addition).

### Static cell culture

A549 cells were maintained inside a T75 flask (Sarstedt, Germany) in a cell culture incubator (37 °C, 5% CO_2_). Cells were subcultured every 3–4 days, when the cells reached 80% confluence. For QuasiVivo experiments, coverslips were sterilized with 70% ethanol and inserted into a 24-well plate. After ethanol had evaporated, each well was washed twice with sterile 1 × Dulbecco’s phosphate buffered saline without magnesium and calcium (DPBS) (Gibco™, United Kingdom). Then, 100,000 cells/well were seeded on each coverslip. Cells were let to attach for either 24 or 48 h depending on the experiment.

#### Collagen coating

For collagen coating, 18.2 µg of collagen (10 µg/cm^2^, for the well area of 1.82 cm^2^) was pipetted on the coverslips of each well. The coatings were incubated for one hour, excessive collagen was aspirated, and the wells were washed once with DPBS to remove the residual acetic acid.

### Cell culture under dynamic flow

For dynamic cell culture, the cell containing coverslips were transferred into each chamber with forceps and a bended needle. Then, conditioned medium (1 ml) was gently added into the chambers to minimize the shear stress generated during the filling of the system. Chambers were sealed and medium (10 ml) was added to the reservoir bottle. QuasiVivo was moved into the incubator, where the tubes were connected to a pump. While moving QuasiVivo between the incubator and laminar hood, two tubes remained unsealed. To minimize contamination risk, the tubes were pinched with fingers during the transport. After connecting the tubes to the pump, the system was filled at the chosen flow rate (Supplementary Tables S1 and S2). The system was monitored until it was properly filled.

### Assessing cell viability

To determine the optimal flow rate, cell viability was assessed with AlamarBlue^®^ reagent and Live/Dead^®^ kit. Viability was monitored with AlamarBlue daily, and the terminal cell viability was confirmed with Live/Dead kit.

AlamarBlue solution (0.5–1 ml, 10% AlamarBlue in the conditioned medium) was gently added into the chambers and well plates. The assembled chambers (Supplementary Fig. S1) were transferred to the incubator, where the chamber tubes were opened to allow proper gas exchange inside the chambers. AlamarBlue solution was incubated with cells for 2 h. Then, AlamarBlue was recovered and replaced with warmed culture medium (1 ml), and fresh medium (2 ml) was added to the reservoir. QuasiVivo was reassembled and the experiment was continued. From each recovered AlamarBlue sample, an aliquot (0.1 ml) was taken for fluorescence measurement (560/590 nm, Varioskan LUX). Lastly, Live/Dead viability assay was performed by incubating the cells in the assay solution (0.5 ml; 2 μM Calcein AM, 4 μM ethidium homodimer-1 in DPBS). The cells were imaged with Cytation5 imaging reader connected to Gen5™ software (version 3.08) (BioTek^®^, BioTek Instruments, Inc., USA). Filter cubes for green (469/525 nm) and red (531/593 nm) light were used.

### Liposomal uptake by A549 cells

Liposomal uptake by A549 cells was determined in static and dynamic conditions. After assembling the QuasiVivo system, liposomes were mixed with the medium (the final lipid concentration was 166 µg/ml), and the PF-TP-601 pump was turned on at a setting corresponding to a flow rate of 500 µl/ml (Supplementary Tables S1 and S2). The uptake of each liposome batch was determined after 1, 2 and 3 h. The coverslips (200,000 cells/coverslip) were moved from QuasiVivo to 24-well plates, where the cells were washed twice with DPBS, detached with TrypLE (0.2 ml), and suspended with DPBS (0.6 ml).

The samples were analysed with BD LSR II Flow cytometer analyser, with a red laser (633 nm), using FACSDiva Software v8.0. For each sample, the fluorescence, forward scattering (FSC), and side scattering (SSC) of 10,000 cells were measured. A population with singular cells was selected based on the cell size and granularity. Untreated cell samples were used as controls for autofluorescence. Fluorescence overlap with the treated and untreated samples was selected to be 5% (Supplementary Tables S3 and S4).

Effect of free HA on the liposome uptake was studied by seeding A549 cells on a 24-well plate (40,000 cells/well) incubating overnight. The next day, the cells were incubated for 1 h in 500 µl of medium supplemented by free HA (10 mg/ml) (Rooster comb, FUJIFILM Waco Pure Chemical, Japan). After the HA pre-treatment and without removing the free HA, the liposomes (166 µg/ml total lipids) were added to the wells and incubated for 1 or 3 h. The cells were washed and detached as described above and analysed with FACS Canto (Becton Dickinson, USA). Free HA treated and non-HA treated cells in three technical replicates were compared.

### Statistical analysis

Data are presented as Mean ± standard deviation. The Mean fluorescence of each flow cytometry experiment sample was calculated in FlowJo, v10.8.1 for Windows (FlowJo, LLC, Oregon, USA). AlamarBlue cell viability and the Mean fluorescence data were analysed with Microsoft Excel, version 2108 (Microsoft, New Mexico, USA). All the significance analyses were conducted with IBM^®^ SPSS^®^ Statistics for Windows, version 28.0.0.0 (190) (IBM, New York, USA). One-way ANOVA with Tukey HSD post-hoc test was performed to determine whether changing the flow rate, attachment time, or coating generate differing cell viabilities under flow conditions. With the same method, cell uptake differences between chamber 1, chamber 2, and well plate were determined. Student T-tests were conducted to compare the viability results between two combined variables (e.g. 250 µl/min flow rate, comparison between attachment times of 24 h and 48 h). P-values less than 0.05 were considered significant. Graphs were created with Origin 2021b (64-bit), SR2 9.850212 (OriginLab, Massachusetts, USA).

## Supplementary Information

Supplementary Information.

## Data Availability

All the original data is available from the corresponding author upon reasonable request.
