# Supplementary material for: Establishing a simple perfusion cell culture system for light-activated liposomes
Source: Sci Rep. 2023 Feb 4;13:2050. doi: 10.1038/s41598-023-29215-6 (PMC9899206; doi:10.1038/s41598-023-29215-6)
Supplement: Supplementary file 1 — Supplementary Information. [file 41598_2023_29215_MOESM1_ESM.docx]

**Supplementary Information**

**Establishing a simple perfusion cell culture system for light-activated liposomes**

**Eija Ilvesroiha^1,*^, Patrick Lauren^1^, Natsumi Uema^2^, Kanako Kikuchi^2^, Yuuki Takashima^2^, Timo Laaksonen^1,3^, Tatu Lajunen^1,2,4^**

^1^University of Helsinki, Faculty of Pharmacy, Division of Pharmaceutical Biosciences, Helsinki, 00790, Finland

^2^Tokyo University of Pharmacy and Life Sciences, Department of Formulation Sciences and Technology, Tokyo, 192-0392, Japan

^3^Tampere University, Faculty of Engineering and Natural Sciences, Tampere, 33720, Finland

^4^University of Eastern Finland, Faculty of Health Sciences, Kuopio, 70600, Finland

*eija.ilvesroiha@helsinki.fi

**
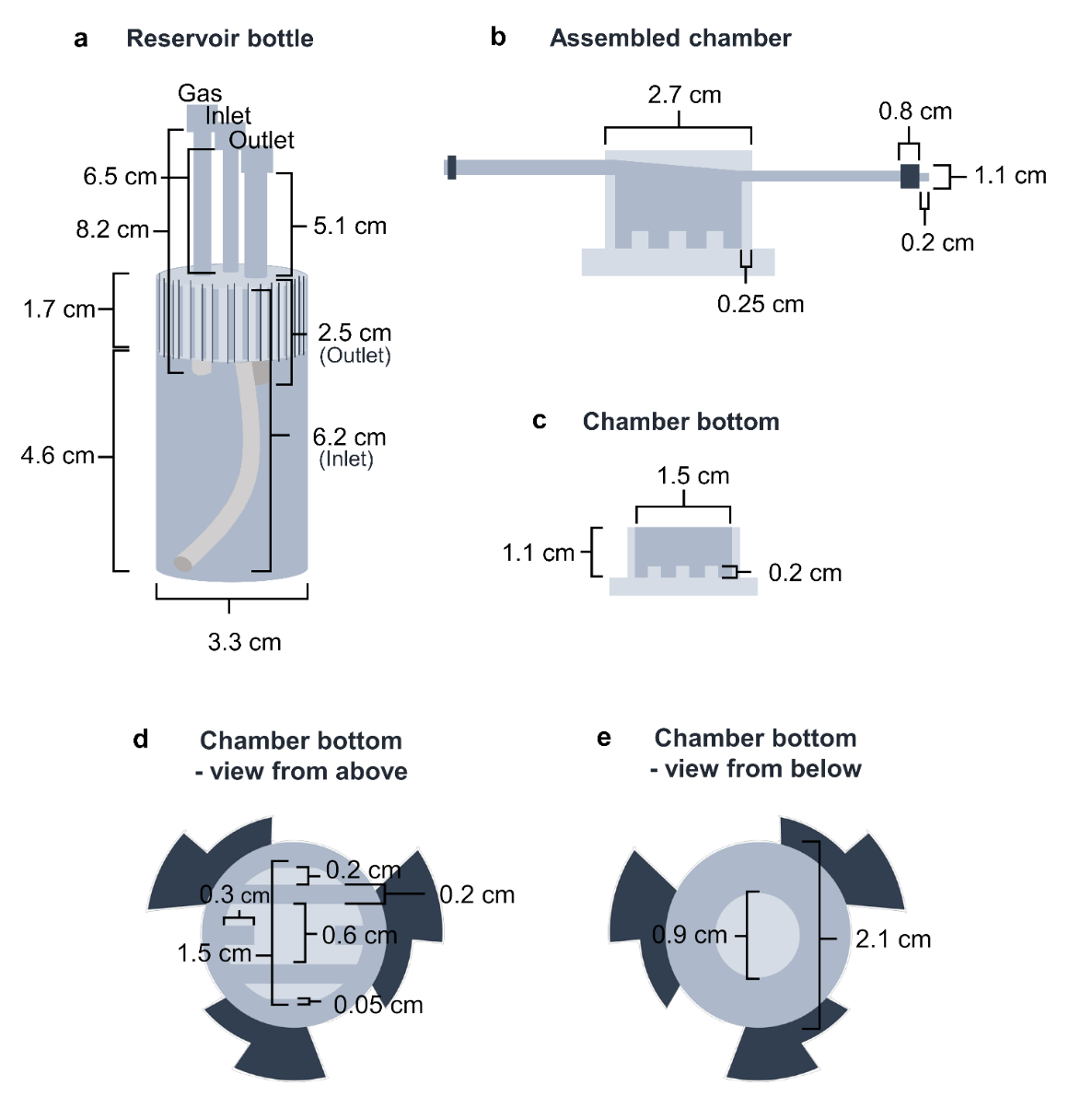
**

**Supplementary Figure S1.** Measurements of separate QuasiVivo system parts. Medium reservoir bottle (a) includes connecting ports for the gas, inlet and outlet tubes. The gas port ensures gas exchange between the cell media and the surroundings. To maintain sterility of the closed system, a filter can be connected into the gas port. QuasiVivo QV500 chambers (b) construct of two pieces, the chamber bottom and the top part. The bottom part (c) resembles a well with grated bottom. Coverslips are placed onto the grate. The tubings of the top part connect the chambers to the system. The outlet tube is higher than the inlet tube to assure proper air bubble removal. The chamber bottom part viewed from above (d) and below (e). The light irradiation was aimed towards the area in the middle, where the material is thinner within the inner circle.

**Determination of the flow rate**

The flow rates yielded by different pump settings were determined following manufacturer’s instructions. The calibration was performed separately for each pump, for PF22X0103 and for PF-TP-601. Specifically, the medium bottle was filled with purified room temperature water (approximately +22 ˚C), the pump was turned on and allowed to stabilize for 10 minutes. Air bubbles were removed mechanically from the system. Then, the flow rates were determined at five settings (1, 3, 5, 7, and 10) by weighting the outlet water in the final chamber.

For analysis, the water weight was adjusted with the factor of 997.76 mg/ml (the mass of water per volume at +22 ˚C) and a calibration curve was generated (Fig. S2). By utilizing the calibration curve formula, the estimated settings for flow rates of 125, 250, 500, and 750 µl/min were calculated. Each estimate was tested in practice (Table S1 and S2), and the calibration curve was adjusted iteratively, to expedite the flow rate optimization for future experiments.

**
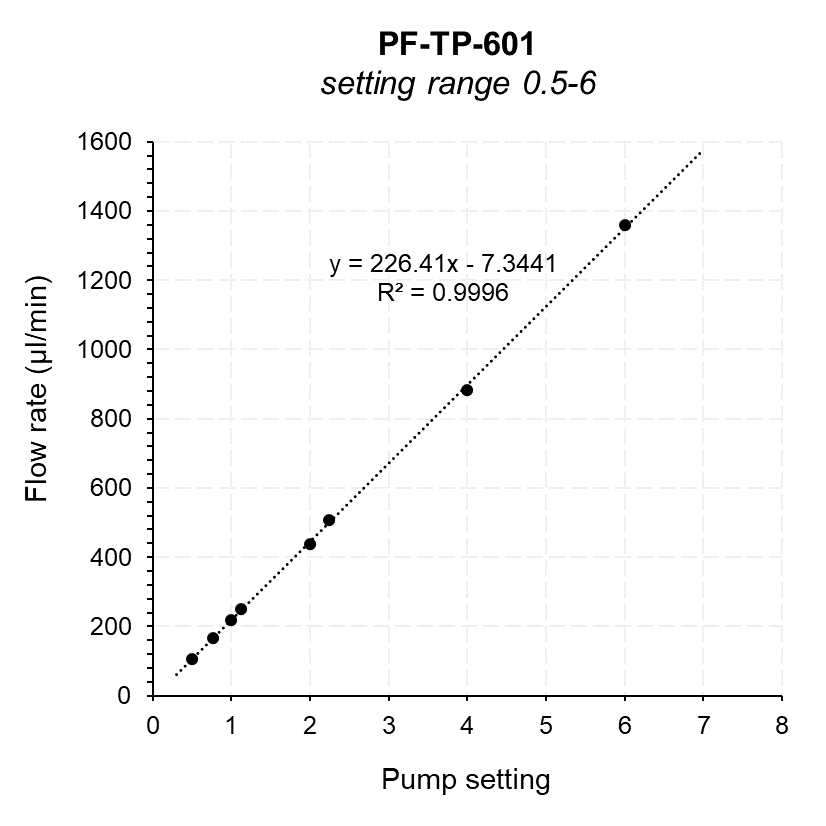
**
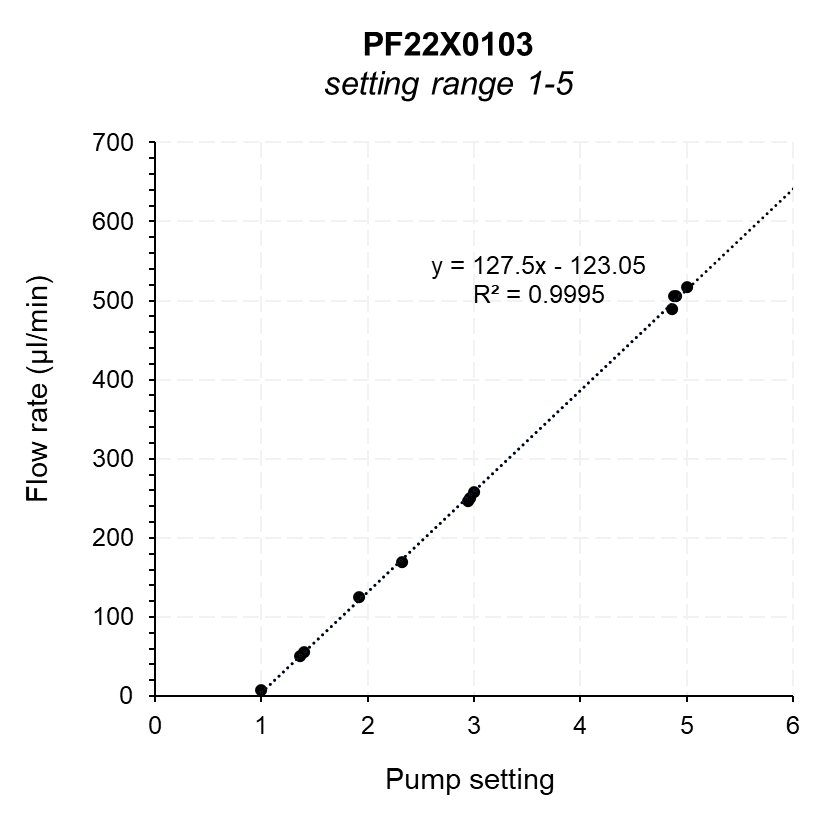

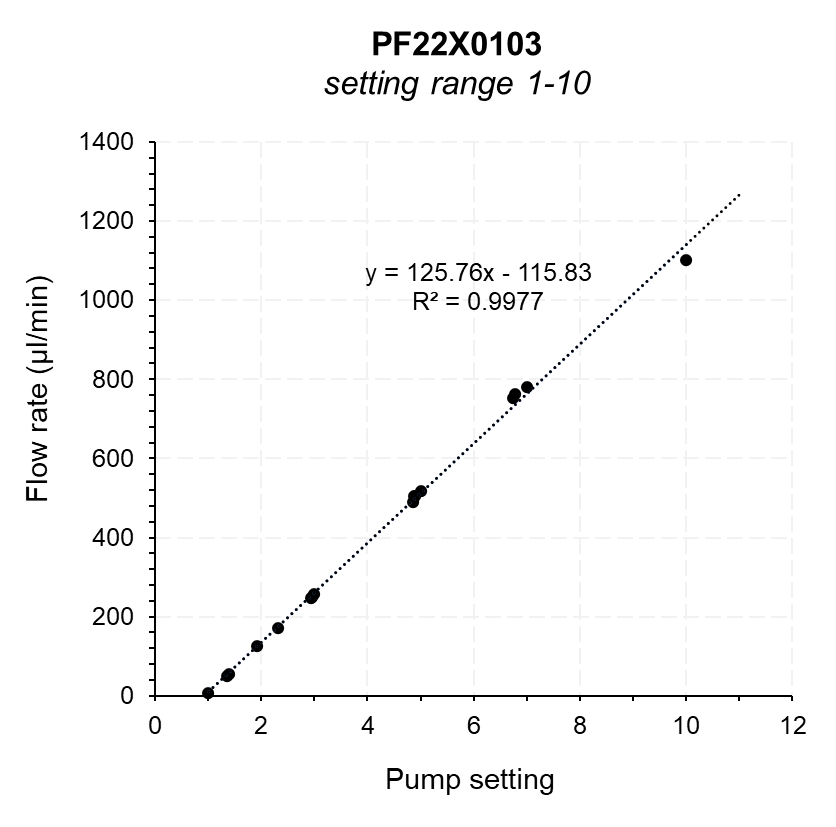


**Supplementary Figure S2.** The calibration curves for different settings. **(a)** Calibration curve created with pump PF22X0103, with settings ranging from 1 to 10 (Mean; n=3). **(b)** Calibration curve created with pump PF22X0103, with settings ranging from 1 to 5. The curve for the shorter setting range was created as the flow rates seemed to correlate more accurately within that range (Mean; n=3). **(c)** Calibration curve created with pump PF-TP-601, with settings ranging from 0.5 to 6 (Mean; n=3-4).

**Supplementary Table S1.** The measured flow rates generated by the QuasiVivo system and PF22X0103 pump (Mean ± s.d., n=3).

**Supplementary Table S2.** The measured flow rates generated by the QuasiVivo system and PF-TP-601 pump (Mean ± s.d., n=3).

**Sterilization of the QuasiVivo system**

To sterilize the QuasiVivo system, the system was first sprayed and then filled with 70% ethanol. After ethanol had circulated within the system for 1 hour, it was replaced with sterile DPBS under cell culture laminar hood. The buffer was removed 24-48 hours later, prior to transferring cells into the system.

**Gating procedure for flow cytometric analysis**

Control cells were first gated based on the cell size and granularity to exclude dead and aggregated cells (Table S3 and S4). Then, out of this population, a gate with 5% fluorescence overlap with the control cells (autofluorescence) was created. This procedure was performed for each separate experiment, always using the control cells (well plate grown cells, no liposomes).

**Supplementary Table S3.** Numerical analysis of flow cytometry data for non-coated liposomes. Cell populations from each experiment are shown.

**Supplementary Table S4.** Numerical analysis of flow cytometry data for hyaluronic acid-coated liposomes. Cell populations from each experiment are shown.

**Particle size of the liposomes**

The particle size of the prepared liposomes was determined with dynamic light scattering method (Zetasizer APS, Malvern Instruments, United Kingdom). Each liposome sample was measured thrice at +22 ˚C.

**Supplementary Table S5.** The particle sizes of non-coated and HA-coated liposomes used in the cell uptake experiments under dynamic conditions. The sizes are shown as an average of three technical replicates (Mean ± s.d.).

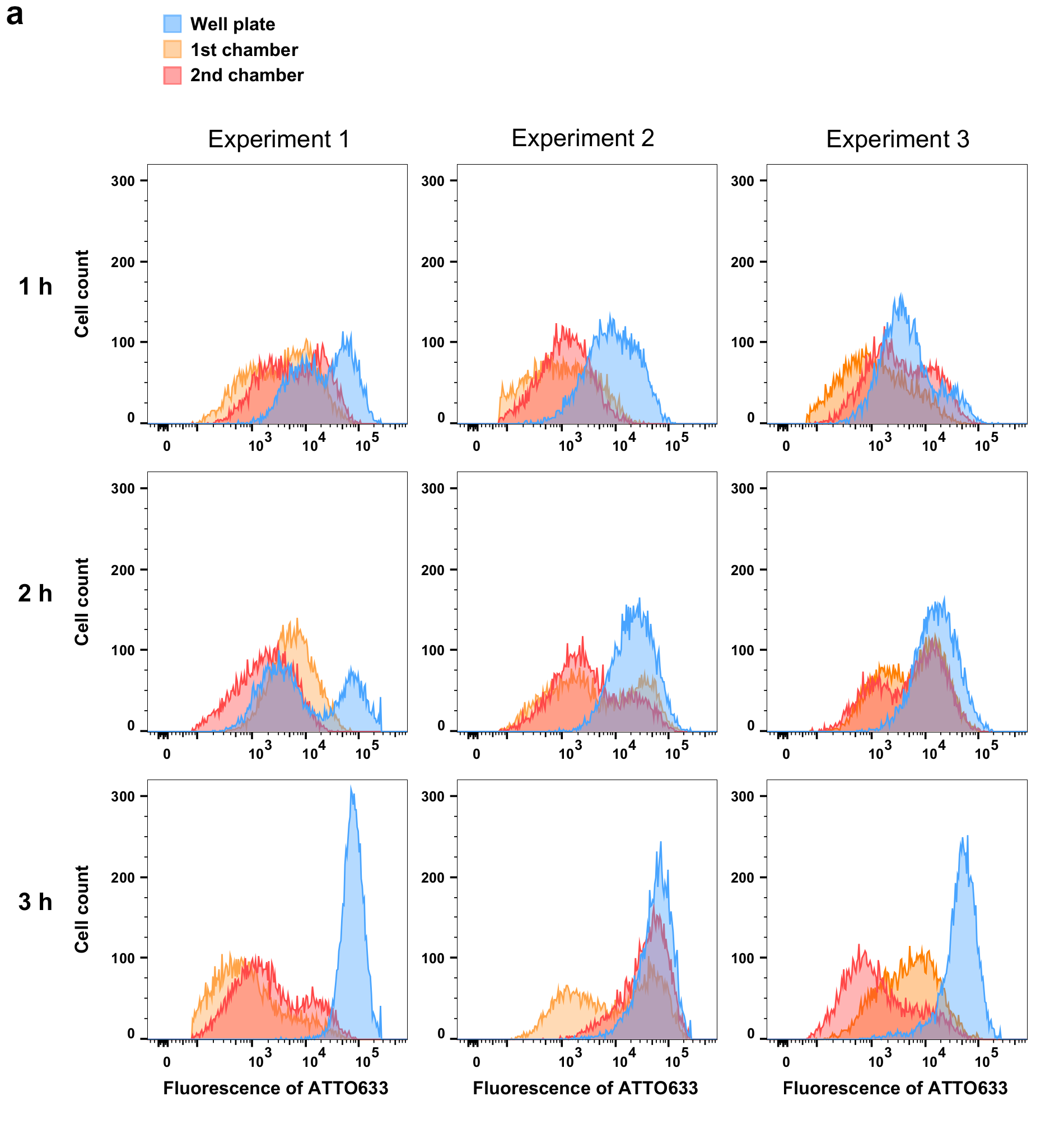


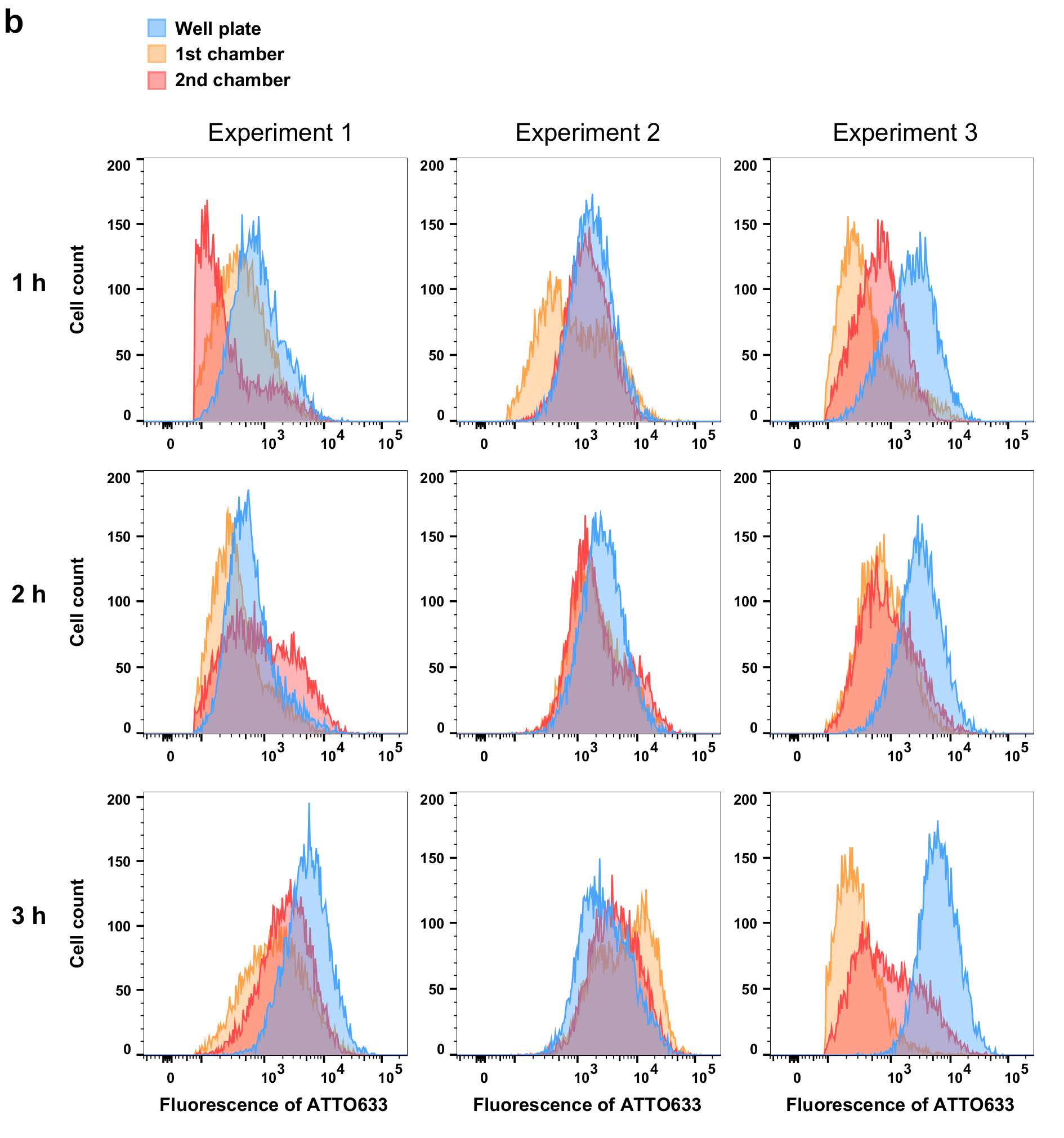


**Supplementary Figure S3.** The fluorescence intensity graphs of cell uptake experiments presented in the main article with non-coated (a) and hyaluronic acid-coated (b) liposomes. Only the liposomal containing samples are shown. Each experiment is shown under each column, and the incubation times are on the horizontal rows. Graphs were generated with FlowJo (FlowJo, LLC; Oregon, USA).

**Liposome coating material comparison in static well plates**

Uptake of liposomes with hyaluronic acid (HA), polyethylene glycol (PEG) coating, or without a hydrophilic coating (NO) by A549 cells was determined in static well plate conditions. The liposomes were prepared as described in the materials and methods section.

A549 cells were seeded on 24 well plates (40 000 cells/well) and incubated over night at 37 °C, 5% CO_2_. Liposomes in growth medium (166 µg/mL of total lipids) were added to the wells and incubated for 1, 2, or 3 hours. The cells were washed twice with DPBS, detached with TrypLE (0.2 ml), and suspended with DPBS (0.6 ml). The samples were analysed with FACS Canto (Becton Dickinson, USA) flow cytometer analyser, with an APC-A channel voltage 500. For each sample, the fluorescence, forward scattering (FSC), and side scattering (SSC) of 10 000 cells were measured. A population with singular cells was selected based on the cell size and granularity. Untreated cell samples were used as controls for autofluorescence. Fluorescence overlap with the treated and untreated samples was selected to be 5%.

Two-tailed Student’s T-tests were performed to determine whether surface coating generates differing nanoparticle cell uptake. The data was analysed with Microsoft Excel, version 2108 (Build 14326.21200) (Microsoft, New Mexico, USA).

**Supplementary Figure S4.** Additional results from a liposomal uptake study under static conditions. This experiment was performed separately from the experiments shown in the main article. Mean ± SD (error bars) reported. For the NO and PEG samples n=2, and for the HA samples n=3. NO = non-coated liposomes; HA = hyaluronic acid-coated liposomes; PEG = polyethylene glycol-coated liposomes; ** p < 0.01; ns = not significant.

**FT-IR analysis of the DSPE-hyaluronic acid conjugate**

To confirm the DSPE-HA conjugation, a Fourier-transform infrared (FT-IR) spectroscopic measurement was performed (Supplementary Figure S5). The analysis was performed with Bruker Vertex 70 FT-IR spectrometer equipped with an ATR crystal. Background was measured 256 times. Then, the transmittance was scanned for both samples 256 times between 600 to 4000 cm-1 with a 4 cm-1 resolution. The data was plotted in Microsoft Office Excel version 2202, build 14931.20764 (Microsoft, New Mexico, USA) and the graphs were compiled in Inkscape version 1.2.


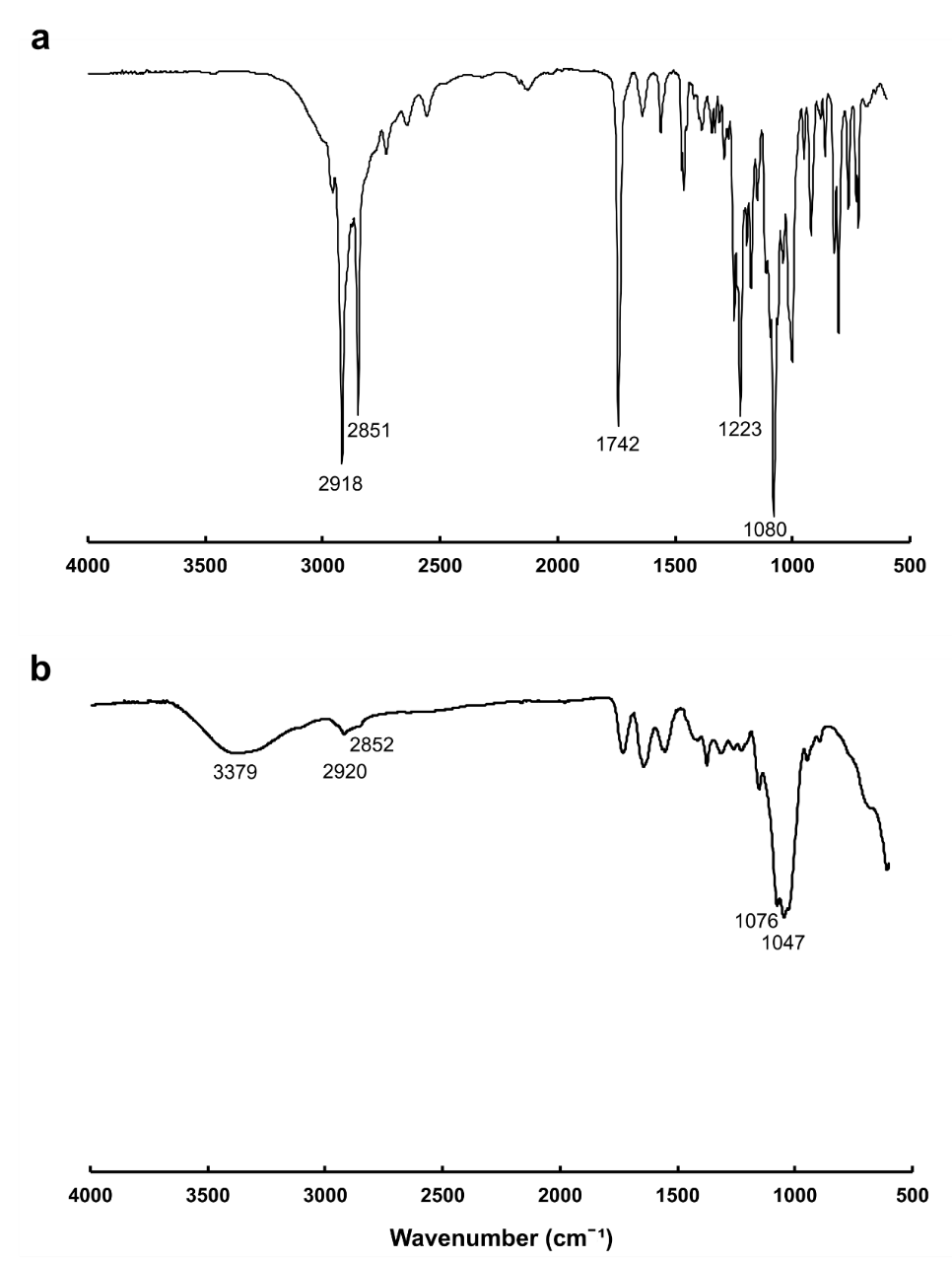


**Supplementary Figure S5.** The FT-IR spectra of DSPE (a) and DSPE-HA (b). **(a)** The C-H bends from stearoyl chains peak at 2918 and 2851 cm^-1^; C=O bends at 1742 cm^-1^; phosphate at 1223 cm^-1^; and C-O bend at 1080 cm^-1^. **(b)** For the hyaluronic acid part, a broad peak for amide is seen at 3379 cm^-1^ and the C-O bends from the hyaluronic acid chain are visible at 1076 and 1047 cm^-1^. The C-H bends from the stearoyl chains of DSPE are also seen at 2920 and 2852 cm^-1^.

**Live/Dead images**

Gen5 generated an automatic contrast adjustment for all the images taken with Cytation5 machine.

**Mycoplasma**

The used A549 cells were tested to be mycoplasma free.
